# Ethical considerations for biobanking and use of genomics data in Africa: a narrative review

**DOI:** 10.1186/s12910-023-00985-y

**Published:** 2023-12-05

**Authors:** Mary Amoakoh-Coleman, Dorice Vieira, James Abugri

**Affiliations:** 1 Department of Epidemiology, Noguchi Memorial Institute for Medical Research, University of Ghana, Legon, Accra, Ghana; 2 2Health Sciences Library, Grossman School of Medicine, New York University, New York, USA; 3 School of Chemical and Biochemical Sciences, C.K. Tedam University. of Technology & Applied Sciences, Navrongo, Ghana

**Keywords:** Biobanking, Genomic research, Genomic data, Ethical considerations, Africa

## Abstract

**Background:**

Biobanking and genomic research requires collection and storage of human tissue from study participants. From participants’ perspectives within the African context, this can be associated with fears and misgivings due to a myriad of factors including myths and mistrust of researchers. From the researchers angle ethical dilemmas may arise especially with consenting and sample reuse during storage. The aim of this paper was to explore these ethical considerations in the establishment and conduct of biobanking and genomic studies in Africa.

**Methods:**

We conducted a narrative synthesis following a comprehensive search of nine (9) databases and grey literature. All primary research study designs were eligible for inclusion as well as both quantitative and qualitative evidence from peer reviewed journals, spanning a maximum of 20 years (2000–2020). It focused on research work conducted in Africa, even if data was stored or analysed outside the region.

**Results:**

Of 2,663 title and abstracts screened, 94 full texts were retrieved and reviewed for eligibility. We included 12 studies (7 qualitative; 4 quantitative and one mixed methods).

Ethical issues described in these papers related to community knowledge and understanding of biobanking and genomic research, regulation, and governance of same by research ethics committees, enrolment of participants, types of informed consents, data collection, storage, usage and sharing as well as material transfer, returning results and benefit sharing. ca. Biospecimen collection and storage is given in trust and participants expect confidentially of data and results generated. Most participants are comfortable with broad consent due to trust in researchers, though a few would like to be contacted for reconsenting in future studies, and this would depend on whether the new research is for good cause. Sharing data with external partners is welcome in some contexts but some research participants did not trust foreign researchers.

**Conclusion:**

Biobanking and genomic studies are a real need in Africa. Linked to this are ethical considerations related to setting up and participation in biobanks as well as data storage, export, use and sharing. There is emerging or pre-existing consensus around the acceptability of broad consent as a suitable model of consent, the need for Africans to take the lead in international collaborative studies, with deliberate efforts to build capacity in local storage and analysis of samples and employ processes of sample collection and use that build trust of communities and potential study participants. Research ethics committees, researchers and communities need to work together to work together to adapt and use clearly defined ethical frameworks, guidelines, and policy documents to harmonize the establishment and running of biobanking and genomic research in Africa.

**Supplementary Information:**

The online version contains supplementary material available at 10.1186/s12910-023-00985-y.

## Background

The era of high throughput sequencing technologies and the rapid growth in bioinformatic algorithms for the manipulation of genomic data has brought with it critical issues in bioethics that are worth considering in the acquisition, biobanking and analysis of genomic data [1]. This calls for clear guidelines to govern the conduct of genomic research and the use of genomic data. Genomic data is a critical resource for the development of novel therapeutics [2]. Sharing genomic data has become imperative for researchers, especially where family data with third parties are concerned For instance, cancer data needs to be shared to fast-track the search for novel therapeutics [3]. There are serious concerns when it comes to sharing genomic data as it carries more information about the participants genealogy and associated risk factors to some diseases [4].

There are important ethical dilemmas when it comes to the genomic research in Africa, with seminal discourse on genomics and ethics in Africa [5]. Participants in Africa must be informed of the use of their data and information generated from such research shared with research participants and their communities. In tackling ethical issues that impinge on genomics research, several attempts have been made at developing robust and carefully thought-out strategies in guiding the informed consent process in genomic research in Africa [6]. Broad consent has been at the center of discussions on ethics in genomic research in Africa [6]. Broad consent would provide ideal grounds for futuristic analyses of genomic data to answer newer questions as they emerge. Although this might be debatable ethically, it assists in overcoming several bottlenecks that may arise at the population level interpretation. Experts in the Africa need to lead the development and implementation of ethical guidelines that govern such details of genomic research, including setting up biobanking and the use of genomic data in future as well as provide platforms for the continuous education on genomic research.

Our study therefore sought to describe the existing ethical considerations for biobanking and genomics research and data use in Africa. Our focus for this review relates to ethical considerations for biobanking and use and sharing of genomic data or stored specimen data generated from work in Africa. This includes knowledge, acceptance, and ethics of biobanking, generation of genomic data, from enrolment of participants (including consenting), sample collection, storage and transport, analysis of the data throughout the entire research process and even beyond for the length of time that the data is archived.

## Methods

We conducted a narrative review following the *preferred reporting items for systematic review and meta-analysis protocols (PRISMA-P) 2015* checklist [7]*.*

### Information sources and search

A medical librarian (DV), trained in systematic reviews, conducted the literature search on September 30, 2020 PubMed/Medline, Embase (Ovid), Cochrane Library (Ovid), Global Health (Ovid), APA PsycInfo (Ovid), Cumulative Index to Nursing and Allied Health (CINAHL), Web of Science, Biosis Citation Index (BCI), and Scielo/Lilacs were the databases searched for bibliographic citations. The World Health Organization and Google were searched for relevant grey literature not found in bibliographic databases. The following journals were searched electronically to ensure articles were not missed through the database search: Bioethics, Bio-preservation & Biobanking, BMC Genomics, BMD Medical Genomics, BMC Medical Ethics, Developing World Bioethics, Genomic Medicine, Human Genomics, and the Journal of Medical Ethics. Search terms included the following keywords: ‘biobank’, ‘biobanking’, ‘biological specimen banks’, ‘biomedical research’, ‘specimen handling’, ‘genomics’, ‘research ethics’, ‘ethics’, ‘ethical’, ‘ethics research’, ‘research ethics committee’ ‘Africa’, and ‘African’. The detailed search strategy is in Additional file 1. All citations were managed through EndNote and uploaded to Rayyan for systematic review management.

### Eligibility criteria

All primary research study designs were eligible for inclusion, including experimental and non-experimental studies. We included studies employing both quantitative and qualitative evidence from peer reviewed journals, spanning a maximum of 20 years (2000–2020). Except for project protocols, reviews, commentaries and reports/conference proceedings, all study designs were included. It focused on research work conducted in Africa, even if data was stored or analysed outside the region. Included articles were all peer-reviewed, written in English and contained the pre-defined domain and determinant and were primary data. The domain for this study was ethical considerations for biobanking and use of genomic data generated from work in Africa. Articles in other language but whose abstracts were available also in English were also reviewed for eligibility. Articles were excluded when they did not match the domain we defined or were reports of conference proceedings or secondary analysis. Reviews and editorials were excluded, but individual studies identified in such reviews and editorials were assessed for their relevance and eligibility based on the above criteria.

### Study selection

All duplicates were manually removed using Endnote. Screening based on title and abstract was done independently by three reviewers for the database searches (MAC, JA and DV). Any discrepancies or disagreements between the reviewers were discussed amongst reviewers until a consensus was reached. Where necessary, full text was assessed for clarity. The authors had access to full text of all included papers.

### Data extraction and synthesis

MAC and JA independently conducted data extraction from the included papers, with no blinding to the journal or author details, using a standardised data extraction form based on PRISMA-P guidelines. First, data on the overview of the characteristics of the included studies was extracted. This included data on the variables author (year), study design, setting (country, population, sample size), study objective, specific genomic issue studied (general biobanking, genomic data), specific body tissue(s) mentioned, specific ethical issue studied, and main findings were extracted (Tables 1 & 2).

**Table 1:** A summary of the included studies

Authors (Year)	Study design	Country (ies)	Population	Sample Size	Objective(s)
Akinyemi (2016) [8]	Case Control study	Ghana & Nigeria	Transnational, multicenter, hospital and community-based study recruited from sixteen (16) sites	6,000 (3,000 cases and 3,000 controls)	To describe our experience with the development of efficient and reliable procedures for collection, processing, storage, and shipment of biological samples across coordinating sites
Abdelhafiz (2019) [9]	Survey (Cross sectional)	Egypt	3 University hospitals from 3 geographic regions of Egypt	210 (259 recruited)	To assess the knowledge, attitude, willingness of Egyptian patients to contribute samples towards biobanks; to assess relationship between knowledge and attitude; to assess the factors and fears that might discourage participation
O’Neill et al. (2016) [10]	Case Study Ethnographic research In-depth interviews, participant observation, informal conversations, and group discussions	Gambia	Village inhabitants in one village out of twelve in rural Gambia	30 (15 men and 15 women)	To explore anxieties around blood-taking during screening for a malaria treatment trial in the Gambia
Schalkwyk et al. (2012) [11]	Qualitative study Semi-structured interviews	South Africa	TB Research participants	20 (16 female, 4 male)	To provide a preliminary exploration into the views of research participants on sample storage and re-use
Tindana et al. (2012) [12]	Rapid assessment using Qualitative methods (in- depth interviews, focus group discussions and observations)	Ghana (Kassena-Nankana District in northern Ghana	Participants in the MalariaGEN project	84 (research scientists and assistants, mothers)	To identify issues arising in practice during the enrolment of paediatric cases with severe malaria and matched healthy controls into the MalariaGEN study
Moodley and Singh (2016) [13]	Qualitative study In-depth qualitative interviews	South Africa Western Cape, Gauteng and Kwa-Zulu Natal (South Africa)	Researchers (medical and scientific researchers, biobank and governance experts)	21	To explore perspectives of researchers working with bio specimens and/or biobanks in South Africa
Tindana et al. (2014) [14]	Qualitative study In-depth interviews and focus group discussions	Kenya Ghana	Researchers; fieldworkers; research assistants; laboratory staff; RECs members; directors of research institutions; community representatives	19 individuals (15 men, 4 women) 3 groups (averagely 6 individuals each)	To identify practical ethical issues arising in the collection, export, storage, and reuse of human biological samples in the context of international collaborative biomedical research
Igbe and Adebamowo (2012) [15]	Focus Group Discussions (FGD)	Nigeria	Adults from different ethnic, age and socio-economic groups	123	To explore the perspectives of Nigerians on donation of specimen for the biobanking research
Mwaka & Horn (2019) [16]	Cross-sectional Study	South Africa (3 universities and 2 research institutions)	Researchers (investigators, scientists, clinicians, pathologists, laboratory or repository personnel and managers who design and implement biomedical research)	400 (62 respondents)	To explore researchers’ perspectives on ethical issues of biobanking, particularly informed consent and ethics review of research involving human resource repositories
Wendler et al. (2005) [17]	Survey (Cross-sectional study)	Uganda	Adults who consented for their children up to 12 years to participate in an RCT	347	To explore views of Ugandans on stored biological samples
Barchi et al. (2015) [18]	Qualitative study FGDs	Botswana	Members of Health research development board (HRDB) and institutional review board (IRB)	41	To explore the knowledge, concerns, and training needs of IRB members in Botswana with respect to the use of biospecimens in biomedical research
Moodley et al. (2014) [19]	Mixed Methods Semi-structured questionnaire with both quantitative and qualitative questions	South Africa	Previous research participants	200	To explore the issues of sample collection, storage, export and future use of stored biological samples

**Table 2:** Summary of bio-genomics and ethical issues studied and main findings of included studies

Authors (Year)	Specific Genomic Issue studied (General, biobanking and the use of genomic data)	Specific Body Tissue(s) involved	Specific Ethical Issue (Including Ethical Guidelines and frameworks elicited/ used)	Main Findings/ Conclusion
Akinyemi (2016) [8]	Genetic and environmental factors that interact to produce the peculiar phenotypic and clinical characteristics of stroke as seen in people of African	Blood, serum, plasma, buffy coat, DNA, red cell concentrates	Informed consent, Material processing and transfer, Governance,	Consent to participate also meant consent for storage of samples Standard operating procedures (SOPs) for sample collection and Material transfer agreements (MTAs) for shipment of samples, were developed and complied with by all sites The project has enormous biospecimen resource for future genomic research stored with the H3Africa biorepository, in compliance with H3Africa guidelines Frequent visits and effective communication channels established Data and biospecimen from the project are freely and widely available within the framework of safeguarding participant safety and confidentiality
Abdelhafiz (2019) [9]	Governance of Biobank	Blood or tissue samples	Informed consent, Confidentiality Community participation (benefits and barriers)	There was scanty knowledge about biobanking among Egyptian patients Many had positive attitude towards sample donation with no religious or cultural barriers. There was no significant relationship with willingness to donate samples and sociodemographic factors Most believed there were benefits in participation but had concerns with sharing their samples across borders or with pharmaceutical companies. They wanted their information to remain private and confidential. Law enforcement agents must have access to their data when necessary
O’Neill et al. (2016) [10]	Sample Collection for research	Blood	Participation (perceptions about donating blood for research) Informed consent	About 42% of inhabitants accepted having a bloodspot taken to screen for malaria Rumours play a key role in anxiety. Although trial recruitment was initially high in the village, some families refused screening when rumours started spreading that the trial team was taking too much blood Women are more vulnerable to weakness after blood donation Some thought blood taken for research would be donated for therapy They believed they had increased nutritional need after donating samples to replace lost blood Concerns about ‘loss of blood’ were equated to loss of strength and lack of good food. Loss of blood can lead to fainting and weakness which will not allow them to work on their farms
Schalkwyk et al. (2012) [11]	Sample storage and re-use	Blood, Saliva, Sputum	Informed consent, Community benefit, Keeping participants informed	Participants expressed a wide and complex range of views about issues of sample storage and re-use and consent regarding these Majority supported one time consent for storage and reuse of samples They generally had limited understanding of genetic research and were not in favour of “for-profit” research They demonstrated a great deal of trust in researchers and reuse of samples. However, they indicated that certain types of re-uses were more acceptable than others
Tindana et al. (2012) [17]	Enrolment of participants into genomic research (health facility and community) Consent for genetic and genomic research	Blood	Community engagement Informed Consent	Education of subjects on genomic studies was better during community enrolment than hospital enrolment. It was difficult explaining genomic research in local languages Participants and staff seeking consent were less aware of the methodologies employed during genomic research and their implications, such as the breadth of data generated and the potential for future secondary research Community engagement processes involved fewer women, so meetings were intentionally organised for women groups
Moodley and Singh (2016) [13]	Biobanking,	Blood Cells Tissues Organs	Competence of research ethics committees (RECs) Governance, Informed Consent, Export and Material Transfer Agreements (MTAs), Community engagement	Researchers articulated serious concerns over standardised regulatory approaches that failed to consider the heterogeneity of biobanks Guidelines and Research Ethics Committees (RECs) need to stratify risk accordingly and governance processes and structures must be flexible While broad general consent was preferred, tiered consent was thought to be more consistent with respect for autonomy and building trust Material Transfer Agreements (MTAs) were often lacking when bio samples were exported, and this was perceived to impact negatively on trust. Authentic community engagement would help to build trust
Tindana et al. (2014) [14]	Export, storage and reuse of human biological samples	Cells, tissues, organs, blood, and sub-cellular materials such as DNA	Informed consent Research governance Sample export	Participants emphasised the importance of sample export, storage, and reuse, and acknowledged the existence of some structures governing these research practices There is the need for several practical ethical concerns to be addressed to ensure high standards of practice and to maintain public confidence in international research collaborations. These concerns relate to obtaining culturally appropriate consent for sample export and reuse, understanding cultural sensitivities around the use of blood samples, facilitating a degree of local control of samples and sustainable scientific capacity building
Igbe and Adebamowo (2012) [15]	Biobanking (sample donation)	Biological Sample -Blood	Informed consent Benefits from research	Participants had limited knowledge of the concept of biobanking but accepted it once they were educated about it Half of study participants supported use of broad consent; a quarter supported restricted consent while the remaining quarter were in favour of tiered consent Most support shipment of their samples to other countries for further research, but they prefer those collaborations to be done only with competent, ethical researchers and they would like to receive feedback about such projects. Participants emphasized the need to ensure that donated samples were not used for research that contradicts their religious beliefs Majority of respondents preferred health care as a benefit from participation, particularly for any unexpected condition that may be discovered during the research instead of financial compensation
Mwaka & Horn (2019) [16]	Biobanking	Tissue, organs, blood and genetic material	Informed Consent Ethical review Regulation	Generally, the attitudes of researchers on informed consent and ethics review of biobank research were mostly ethically well informed, expressing opinions that were in line with national guidelines. Researchers were unanimously in agreement that issues concerning informed consent are very crucial in biobank research and require considerable discussion during ethics review process. They opined that broad consent is acceptable to biobank research and research participants should have the right to establish acceptable limits on the utilization of their samples for research
Wendler et al. (2005) [17]	Storage and re-use of samples	Blood	Informed Consent	Most of the respondents were willing to provide samples that had codes which can be traced to their children, while future use of samples would require IRB approval Most respondents were willing to share their samples with local and international researchers for future research They were also willing to have samples used for any research
Barchi et al. (2015) [18]	Collection, use and storage of Biosamples for research	Human Biological Material	Informed Consent, Privacy and confidentiality Export of specimen Potential benefits; Regulatory guidance	Culture, norms, and beliefs play a role in community perspectives on biospecimen collection and use in research Belief systems about bodily integrity and strong national identity in the construct of benefits may be at odds with initiatives that involve foreign biorepositories There is lack of understanding among patients and providers about the use of biospecimen in clinical care and research; reuse of biospecimen, particularly issues of consent, ownership, and decision-making Several respondents were okay with broad consent at the time of sample collection but believe individuals do not fully understand such choice Others had concerns about export of specimens and loss of control over reuse and potential benefits were also areas of concern; and felt need for regulatory guidance and IRB-member training
Moodley et al. (2014) [19]	Use, storage, and export of biosamples	Blood	Informed Consent, Confidentiality, Ownership Export,	Most participants were supportive of providing samples for research, but had serious concerns about future use, benefit sharing and export of samples While researchers view the provision of biosamples as a donation, participants believe that they still have ownership rights and are therefore in favour of benefit sharing Almost half of the participants expressed a desire to be re-contacted for consent for future use of their samples 25% of participants had concerns about sample export

Studies were grouped into three types: mixed methods, qualitative and quantitative (which were surveys or case control study). The data synthesis aimed to provide a narrative analysis of included studies, focusing on the scope of ethical issues related to biobanking or research using biomedical sample. A qualitative synthesis of information from the included studies was conducted with studies analysed according to main themes that emerged from the ethical issues discussed. Due to heterogeneity of the few included quantitative studies (designs, settings, outcomes), we were unable to group the results together to conduct a meta-analysis for an overall quantitative conclusion.

### Quality assessment of included papers

The quality assessment was conducted by MAC and JA using the mixed methods appraisal tool (MMAT) version 2018. The tool was used because the included studies employed quantitative, qualitative, and mixed methods. Quality of qualitative, quantitative, and mixed methods papers were assessed separately using the relevant sections of the tool.

## Results

### Overview of included studies

**Fig. 1:**
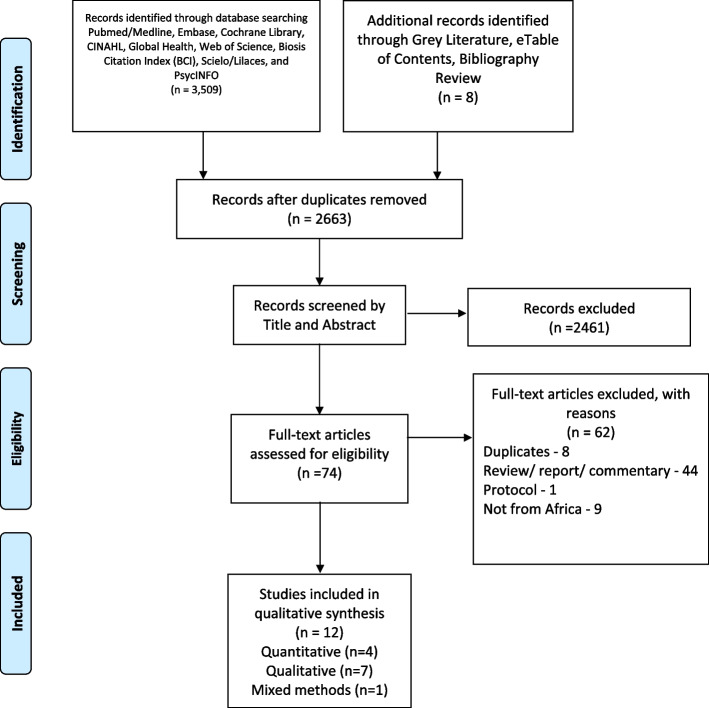
Study selection using the PRISMA flow diagram

A total of 3,517 papers were identified in the database and grey literature searches. After removal of duplicates using Endnote, 2,535 articles remained and were screened by title and abstract. This resulted in exclusion of 2,461 articles, leaving 74 articles to be screened for eligibility. Another 62 articles were further excluded due to the following reasons: eight duplicates, 44 reviews, reports, or commentary, one study protocol, and nine were not studies from Africa. A total of twelve (12) articles were included in our study, four quantitative and seven qualitative and one mixed methods study. Figure 1 illustrates the study screening and selection process. Four studies (33.3%) were conducted in South Africa, and the rest were conducted in Ghana (2), Nigeria, Gambia, Uganda, Egypt, Botswana, and Kenya.

#### Narrative synthesis

We found studies that described perspectives of researchers, previous and prospective study participants and or their relatives or care givers, on ethical issues related to biobanking and genomic data collection, usage and sharing in Africa.

We grouped our results under the following subheadings based on our findings from the included studies, first presenting any quantitative data, followed by the qualitative data:

- Biobanking – knowledge, establishment, participation, regulation, and governance
- Participant engagement and enrolment
- Preferred consent models
- Sample collection and storage, sample reuse
- Confidentiality
- Return of results
- Sample export and benefit sharing

##### Biobanking – knowledge, establishment, participation, regulation and governance

Knowledge on biobanking was associated with higher education and maleness [9], and there was difficulty in explaining genomics in local languages [12]. In one study, researchers found innovative ways to explain essential parts of the term genomics, with parents relating genetics to hereditary characteristics, making it easier to understand [8]. In another study, participants suggested more engagement of the community as a means of strengthening understanding of biological research. For example, organizing an open day for community members to visit laboratories to see the conduct of research would help increase knowledge, awareness, and participation in genomic research [14].

The role of biospecimens in clinical research is often misunderstood. Giving samples for clinical care is considered an act for one’s own wellbeing – leading to treatment for cure [18]. When participants provide biospecimen in the hospital context, they do so, expecting a cure for their condition. Some believe that collection of some special specimen e.g. spinal fluid, worsens health and may hasten death. Due to some of these beliefs, during extraction or amputation, patients ask for their limbs or tooth to take home [18].

Central to setting up biobanks is the role of review and ethics committees (RECs) [8, 13, 19]. Most participants in one study recommended the need to have discussions about standardizing the process of reviewing biobanking research applications [8]. In one study that assessed the competence of research ethics committees (REC) to review biobank projects, researchers distinguished between study specific biobank and biorepository, and noted that RECs must understand heterogeneity of biobanks. It emphasized the need for RECs to stratify their reviews according to risks related to volume and types of specimens since this is important for decision-making and governance of biobanks [13]. The study mentioned that REC members sometimes lack the expertise to review protocols expertly, and that national audit of biobanks and their governance structures is required. International standards and best practices should be followed during biobanking as was done in the SIREN project [8]. Some of these best practices were but not limited to, obtaining individual consent from each participant, development of standard operating procedures (SOPs) for managing samples which were adopted by laboratories at all sites, standardization of sample collection and processing, periodic visits to study sites, effective communication, having material transfer agreements (MTA) for all sample shipments, development of guidelines for accessing biospecimen, hands-on training for staff, regular consultations and consortium meetings, quality control/ assurance measures and use of laboratory information management systems.

With respect to participation in biobanks or genomic research, one study reported 89% (352/396) of respondents believed that study participants have rights with respect to what is done with their tissues, and that these should apply even to anonymized samples [16]. Another study reported between 79% (202/259) – 85% (221/259) of participants willing to donate samples for biobank, some on condition that samples are anonymized, and 53% (151/259) were willing to donate specifically for genetic related research [9]. However, some papers reported refusal of participation or challenges in getting participants [8, 10, 14, 18]. In one study, as many as 62.8% (149/237) potential participants could not be screened for inclusion in a study due to lack of consent by heads of households [10]. Some reasons given for refusal to participate include the fact that blood-taking depletes life-force and body strength which affects health of participants and their capacity to work. Another reason was that it is dangerous for pregnant women and women in general to give blood since they are vulnerable. Some were unable to distinguish between blood sampling and blood donation, believing that some of the sampled blood will be donated or sold to others. Others feared that more blood will be taken than is needed, and that taking blood is associated with hospitalization, with subsequent need for transfusion which has financial implications for them. Other challenges were identified as barriers to participation. Barchi reports that cultural traditions, norms, and beliefs around human tissue and its meaning compelled participants to compare donation of human biological material to practice of male circumcision, in which case the tissue may be sold to others for use. Others also believe that their samples may be stolen by others from the health workers for magic to harm or bring benefit to others [18]. One study also identified local cultural sensitivities around the use of blood samples, with apprehension about blood more than other samples (such as urine, stool, saliva), and export and storage of the samples. Concerns such as pain for children and the volume of sample causing harm to sick children and making them weaker were also reported. There were also issues of mistrust with rumours of researchers selling some of the blood (due to the idea of transport of samples and transfusion) [14].

Regarding regulation and governance of biobanks*,* some studies reported challenges [8, 13]. Researchers mentioned RECs having issues with the objectives of study protocols, informed consent and documentation as well as the need for additional ethical reviews for new studies or lack of plans to deal with community harm/ benefits, as challenges [8]. One study highlights infrastructural and security needs, especially power interruption which affects freezers, the quality of samples and retrieval systems [13]. Lost samples are a violation of promises made to participants. Sustainability of biobanks requires stable and continuous funding to avoid wasted samples which undermines the trust of participants. The study also mentions that multiple levels of governance are imperative in biobanking because biobanks usually involve different institutions and multi-tiered governance systems with varying legal and policy frameworks [13]. In one study, some participants (researchers) were critical of existing regulatory systems, indicating that they are not clear on biobanking, do not have a proper definition of tissue, are often written for therapeutic biobanks and not research biobanks [14]. The study discussed the importance of local capacity building and effective research governance, recognizing the point that while international collaboration is important for scientific research, it works well on mutual trust, transparency and respect and scientific leadership [14]. The use of more protective measures and assurances that allay concerns are needed. Capacity building should be focused on technology and infrastructure training and retaining local personnel with requisite skills to contribute to the conduct analysis and reporting of research locally. The paper concludes that effective research governance structures must be central to the gatekeeping role of RECs, and, institutional and national guidelines should be in place to govern research practice, with RECs serving as Trustees of research samples.

##### Engagement and enrolment of study participants

Two studies identified the importance of stakeholder (community and patient) engagement for biobanking and genomic research [12, 13]. One discusses a model of engagement based on traditional practices that had been established in their study community and followed by all researchers. This involved consultations with the gatekeepers of the community (chiefs and elders), followed by community durbars with the wider community [12]. Enrolment of cases in the hospital also involved a two-step process of consenting, first at the time of admission, and subsequently a second consent was sought based on eligibility criteria. Enrolment of community participants involves a traditional multi-level process which engages heads of compound and household to seek their permission, and then parental consent. The second study also mentions community engagement as a priority to build trust and encourages that it should be undertaken extensively using community newspapers, educational videos advocacy groups that represent trust, and encouraging community feedback [13].

##### Preferred consent models

*Who to consent*

One quantitative study reported on who should give consent in studies involving children [16]. In the study which had researchers as respondents, 84% (317/396) of them agreed that parental consent is enough to store child genetic sample for children who cannot assent because they do not understand the nature of the research, while 92% (364/396) thought once the child understands he/she should give assent. A total of 45% (178/396) of participants believed children between 16–18 years, can understand implications of storage of samples for future research, and that they should have the right to withdraw from the study once they reach age 18 years.

On type of consenting, different models were preferred from the included studies.

*Broad consent*

In one quantitative study, 66% (262/396) of participants preferred broad consent, and this was irrespective of whether participants had ever participated in biobank research [16]. A couple of qualitative studies showed preference for one-time broad consent [11, 13, 15]. One such study which describes broad, tiered, and dynamic consent models, indicated that most researchers preferred one-time broad consent because participants saw reconsenting as impractical and resource intensive, and some found detailed consenting not to be sustainable for clinician researchers [13]. Community researchers, however, found broad consenting unacceptable to their clients and prefer reconsenting whenever necessary. A qualitative study reported that half of respondents supported broad consent, saying they donated samples because they had faith in the research [15]. One-time consent was also perceived as good if all future re-use is listed from the onset, otherwise previously undescribed use should seek reconsent [11]. In a study in which 58% (230/396) of participants did not think reconsent is necessary, reconsent was deemed necessary in a variety of scenarios such as investigating unrelated condition (65%, 257/396)), or when researchers want to add other genetic measures to study (67%, 267/396) [16].

Another paper listed that although blanket consent may be given, participants may not fully appreciate the risks and potential benefits, especially from vulnerable populations [18]. Consent for reuse is mostly preferred, after IRBs approve the reuse.

*Tiered and dynamic consent*

A tiered consent model was described to include specific and broad consent which is enabled and facilitated by technological advancement, while dynamic consent and participation, facilitated by constant information sharing through technology was also preferred by some in one study [11, 16]. One study found 49.5% (99/200) of participants wanted to be contacted for future use of their samples, even if an IRB approves the new use. They didn’t think RECs can consent on their behalf. The rest were comfortable with broad consent to allow re-use of samples [19].

*Reconsenting*

In one study, researchers found it difficult explaining future uses of blood at the point of collection, and acknowledged that requirements of full disclosure cannot be assured in broad consent [14]. It argues that though reconsenting is difficult, it should be done through community engagement, moving away from blanket consent since it limits acceptability of research and defers future consenting to local ethics committee. This is corroborated in another study where most participants (82%, 324/396) thought RECs approval was enough if re-consent was not possible [16]. The pparticipants were evenly split on whether reconsent is necessary to share de-identified samples with another investigator.

*Content of informed consent form (ICF*)

Researchers were also concerned about the actual information on the informed consent form (ICF), saying it is researcher and legal—focused but may not be important for the patient/ community. One study describes timing of consent, with parents satisfied with timing of consent and two-steps process of consenting [12]. The timing of the consent takes into consideration the emotional state of the mother before approaching her for consent. The optimal time was found to be between 24 to 36 h after admission of their children. The two-step process of consenting entailed first explaining the process at the community level, Then when mothers have their children admitted to the hospital and meet the eligibility criteria, they are then specifically enrolled into the study.

##### Sample storage, ownership and sample reuse

*Storage*

Four papers described considerations around storage of biospecimen [11, 17–19]. In one quantitative study, 95% (335/353) of study participants were willing to have their child’s sample stored with a code linked to a patient identifier [17]. In two studies, most study participants were unconcerned about storage of their samples for future use, believing the samples were no longer theirs once it’s given [11, 19]. Participants wanted their samples to be stored securely and used for the purpose for which they were taken, being accessible only to research staff [11]. In one study, many participants felt that specimen belonged to donors, who should retain rights to them including storage, and retrieval if needed, possibly with gradual transfer of ownership to researchers [18]. Some had concerns about storing specimen beyond what is used for care; finding it worrisome that some sample is kept for another purpose. The study recommends the need for a regulatory framework to safeguard storage and duration of same, and the need to build capacity in-country for storage, safekeeping, and research, recommending that specimens belong to Botswana/government and should not be exported [18].

*Ownership*

Only a few believed they still were owners of the samples, with 12% (24/200) wanting a reason to grant permission of sample storage [19]. In one study 50% (130/259) believed the sample does not belong to donor once it is given (8).

*Reuse*

Multiple uses of biospecimen and data is common with genomic research and some studies reported on use and sharing of the biogenetic specimen. In one quantitative study, few (4%, 14/353) participants feared researchers might sell their samples or use them for other non-research related purposes [17]. Most (97%, 343/353) wanted to know about future studies prospectively, 85% (300/353) were willing for their samples to be used for HIV studies and 81% for any disease. The majority (95%, 335/353) were willing to share their samples with researchers in Kenya and Tanzania as well as UK and USA. Living in peri urban areas was associated with being more likely to believe study samples would be used for research purposes only and wanting information about studies [17]. A study in Ghana found that the community does not appreciate data sharing, and that having a policy allows data to be shared with external researchers [12]. Sharing and reuse of data or samples were acceptable by participants if there was a clear data release policy, if new research was for a good cause or would come up with new health solutions and if revenue generated from the research will be shared with them [12]. Good cause refers to current and future community benefits from the study, academic and institutional benefit, and career advancement. Commercial purposes were not considered as good cause [11].

In respect of access to stored samples, priority should be given to local researchers to access samples to benefit Botswana. Some families wanted their samples to be kept identifiable so that they can reconsent when needed for reuse. It should also be possible to link specimen to new discoveries relevant to health. Some international need for reuse may not align with local needs [19].

##### Confidentiality

One quantitative study reported that most participants (91%) in biobanks want researchers to maintain privacy and confidentiality of donor information [9], with 70% believing this will be done. More than half (64%, 166/259) believe data collected will not be used for other reasons without their consent, with 72% believing that law enforcement agencies can have access to their data when necessary.

##### Return of results

Two studies reported on return of results to study participants. Fifty-five percent (142/259) of participants in one study wanted results of tests conducted on their samples to be put in their medical records and 93% (240/259) of them want to be contacted if their results show any risk [9]. In another study, the majority wanted to be informed about their individual results to know their health status as well as benefit from new discovery. Feedback to study participants should be preceded by counselling. A few did not want to know test results because of fear [15].

##### Sample export and benefit sharing

Some studies reported on concern over sample exports and who eventually gains from these samples [9, 13–15, 19]. Some challenges were the loss of control over use of samples and data once transferred, local researchers’ inability to account for exported or shared samples or data, possible use for other purposes and analysis unknown to local researchers, fear of use for rituals, lack of recognition of authorship of local researchers in future work using samples, and others taking credit without acknowledging researchers or community. Participants did not want their samples to be taken to the UK, USA, Europe, and Israel in particular, which is an “enemy of Muslims” [19]. Due to concerns about sample exports, local researchers should ensure local control of samples during and after transfer and scientific collaborations should be supported by mutual agreements, including material transfer agreements [14]. Participants (laymen) in one study were willing to share their specimen and data with commercial and non-commercial entities [15]. A few wanted to be contacted by any new researchers, concerned that if samples were sent outside Nigeria, findings will be used to discriminate against Nigerians or used for something against their religion. Also, collaboration should be with only competent institutions/researchers and feedback would be required [15]. In a qualitative study, strong opinions were expressed on export and material transfer agreements [13]. In this study, researchers insisted that patients must be told at the onset that their samples would be sent abroad with contracts. It is important to prevent unilateral transfer of samples out of Africa and to retain intellectual rights here. The lack of a national MTAs and export permits was of concern. Local collaborators and participants exhibit a lot of trust, but sometimes external partners are not respectful and are surprised that a MTA is asked for. The H3 Africa project biobank will provide specimens to researchers anywhere with an approved protocol. Movement of samples within Africa could also be problematic as some countries do not have MTAs [13]. In the study in Ghana, research ethics committees were also concerned about exported samples due to inability to control what is transported [14]. MTAs are now coming up, and projects should build local capacity to analyse samples locally and thus reduce the need to export samples. Possibly local research institutions should be established to take responsibility and update research ethics committees on overseas analysis, to make external partners accountable.

*Benefit sharing*

In one study, 25% (64/259) of participants believed that donors should be compensated financially [9]. Another study assessed the risk–benefit ratios of biobanks and emphasized that individual benefit is distinguished from public health benefit [13]. Most participants were confident about scientific and clinical benefits of biobanks but felt individual potential benefits are lost due to anonymization of study samples. Long and short-term benefits must also be distinguished, realizing that biobanking is for future benefit and must be done in such a way that retrieving data is not jeopardized. Some risks identified included over-researched communities, with different project groups competing for the same participants in the community; commercialization where samples are sold to pharmaceutical companies; infectious disease samples being a risk to biobanks and patients and so the need for robust infection control. Stigma associated with genetics and genomics making participation by some patients difficult was also mentioned. In a Ghanaian study, participants believed that research is for the benefit of participants, and that participants unmet needs and parents’ expectations of free medical care for their children is a motivation for their participation in such research, and other studies in the community had offered such benefits [12].

Still on potential benefits and benefit sharing, IRB members in Botswana believe subjects should continue to be paid for their time in the study, and any benefits from their stored samples – monetary, intellectual property rights, new treatment – should be shared with the participants, communities, and the nation at large [18]. They were worried that if samples are stored outside, they would be denied these benefits. They also agreed that not adequately acknowledging research subjects and lack of feedback to subjects (including results), affects others’ participation in research later. Some participants (40%, 79/200) mind if profit is generated from work with their samples and 43% of these want to share in the profit. Some 20% (39/200) do not mind if profit is generated for a good cause [19].

### Quality of included papers

In all, we included 12 papers, comprising of seven (7) qualitative studies, four (4) cross sectional studies and one (1) mixed methods study.

For the qualitative studies, all except two of them had clear research questions and the collected data addressed the questions; for all studies, the qualitative approaches and the data collection methods employed were appropriate to answer the questions. The results, and interpretations of same, were adequately derived from the data collected, and there was coherence between the data sources, collection, analysis, and interpretation.

For the cross-sectional studies, 75% of the studies had clear research questions. For all the studies, the collected data addressed the questions, and the sampling strategy was relevant to address the question. Three-quarters of the studies had a representative sample, and all studies used appropriate measurements. The risk of non-response bias for all the studies was not low for 50% of studies and unclear for one study. Statistical analysis was appropriate for all the studies.

The only mixed methods paper did not have a clear research question, and thus it is not clear if the data collected addresses the question(s). Table 3 provides details of the quality assessment.

**Table 3:** Quality Assessment of included studies (*n* = 12)

Author, Year	Are there clear research questions?	Do the collected data allow to address the research questions?	Is the qualitative approach appropriate to answer the research question?	Are the qualitative data collection methods adequate to address the research question?	Are the findings adequately derived from the data?	Is the interpretation of results sufficiently substantiated by data?	Is there coherence between qualitative data sources, collection, analysis and interpretation?	Comments
**Quality of qualitative studies**								
Schalkwyk et al. (2012) [11]	Y	Y	Y	Y	Y	Y	Y	
Tindana et al. (2012) [12]	Y	Y	Y	y	Y	Y	Y	
Moodley and Singh (2016) [13]	Y	Y	Y	Y	Y	Y	Y	
Tindana et al. (2014) [14]	Y	Y	Y	Y	Y	Y	Y	
Igbe and Adebamowo (2012) [15]	N	C	Y	Y	Y	Y	Y	There were no clear research questions stated in the “Introduction”. It was briefly mentioned in the “abstract”
Barchi et al. (2015) [18]	N	C	Y	Y	Y	Y	Y	There were no clear research questions stated in the Introduction. It was briefly mentioned in the abstract
O’Neill et al. (2016) [10]	Y	Y	Y	Y	Y	Y	Y	
**Quantitative descriptive**								
Abdelhafiz et al. (2019) [9]	Y	Y	Y	Y	Y	N	Y	
Mwaka & Horn (2019) [16]	Y	Y	Y	N	Y	N	Y	Out of almost 400 individuals who were sent the survey, only 62 responded and completed it
Wendler et al. (2005) [17]	N	Y	Y	Y	Y	Y	Y	The sample size was not clearly stated
Akinyemi (2016) [8]	Y	Y	Y	Y	Y	C	Y	
**Mixed-methods**								
Moodley et al. (2014) [19]	N	C	C	Y	Y	Y	Y	

Hong QN, Pluye P, Fàbregues S, Bartlett G, Boardman F, Cargo M, Dagenais P, Gagnon M-P, Griffiths F, Nicolau B, O’Cathain A, Rousseau M-C, Vedel I. Mixed Methods Appraisal Tool (MMAT), version 2018. Registration of Copyright (#1,148,552), Canadian Intellectual Property Office, Industry Canada

*Abbreviations:* *Y* Yes, *N* No, *C* Can't tell

## Discussion

Our synthesis of the twelve included studies focused on the ethical considerations of biobanking and genomics research and data use in Africa. This is an important area that needs more exploration because of the paucity of such research in the sub-region and the strong influence of external collaborators in the field. Ethical issues surrounding research and specifically biobanking and genomic research are crucial since informed participation as well as human subject protection must always be assured [20]. All stakeholders in the process must bring their perspectives and experiences to bear in progressively shaping the course of how future research are set up and implemented.

The ethical considerations gleaned in this review are corroborated findings in a previous review [21], and highlight the need to increase awareness and knowledge about biobanking especially in the context of Africa where there many myths surrounding other people having access to one’s biological tissue. Potential study participants need to be well informed about the purpose of biobank-based genomic studies so that their participation will be well informed. Stakeholder engagement and proper community entry that leverages the existing governance structures of communities and their cultural practices have been found to be appropriate and makes communities more receptive when these are adequately carried out with prior planning [5, 8, 13, 22–24]. Community durbars for example, allow for wider participation and afford researchers the opportunity to explain the research to the community, eliminate myths and doubts about the usefulness of genomic research and biobanking to the community, so that they are more likely to partake in such research.

Biobank-based genomic studies should be regulated by both local and national regulatory mechanisms that ensure that participant autonomy, privacy and safety are not compromised in any way. This overtime will help build trust between the public and research communities. Research ethics committees are very central to this process. They are expected to be adequately trained and have expert knowledge to make them effective in addressing all the potential ethical issues that could arise with different studies and prescribe guidance to researchers [13]. They should also be positioned to monitor such research in a consistent manner that will assure public confidence in them as well as for biobanks to serve their intended purposes. There is evidence that regulation of biobanking in the African context is weak, often allowing unethical practices [25]. National level biobanks and genomic data governance structures, that are well equipped to ensure periodic audits of biobanks and biorepositories are required, especially due to international collaboration usually involved in genomic studies in Africa. These governance structures should provide the required oversight responsibility and ensure that biobanks and genomic data are not misused. This strategy would by and large build confidence in research participants and their communities to enable them trust that their samples would be used for their intended purposes.

On the issue of preferred consent models, several models were spelt out by various studies. However, broad consent was arguably the most preferred, with studies reporting the need to be supplemented with reconsenting wherever possible [16]. Where reconsenting would be impossible, RECs approval suffices. The choice for broad consent is based on trust imposed in researchers and possibly RECs to protect participants’ interest throughout the project. Specific future use of biospecimen may not be known by researchers and may be difficult to explain to participants at onset of study. Participants, however, had opinions about what a good cause is for which in future their samples can be used. It is important that RECs and researchers work in sync to ensure that biospecimen are used for approved research purposes, in order not to jeopardize community trust.

Research results, especially genomic data often contains personalized data and issues of confidentiality and return of results arise. Our review shows that participants care about their privacy and trust researchers to ensure confidentiality of their personal information [9]. Any identified risk should be communicated to them in the best way possible. Taken together, it behooves researchers to maintain confidentiality and act responsibly and be judicious in sharing information on risk factors revealed from biobanks and genomic data. Some guidance on return of results of genomic research is available, and these relate broadly to what results to share, who to relay the results and what actions should follow return of results [26, 27].

The existence of international collaboration and partnerships in research involving biobanks and genomic data requires that data is sometimes shared with external partners. Ethics committees should have clear guidance for researchers regarding this process. Research protocols should at the very onset have plans on MTAs, allowing study participants to be informed if their samples will be shared with other countries, and how samples would be used by collaborators. This will allay the fears of samples being used for other ritual purposes in foreign countries [13]. Unfortunately, as reported in one study, sometimes RECs have expressed worries about their inability to control the use of exported samples once they leave the sample origin country [14]. Irrespective of well laid out MTAs, some collaborators were reported to have used samples from biobanks and genomic data for other purposes without recourse to the sample origin RECs. This may not be a disadvantage to only participants but also to the collaborating researchers. Data and results generated by such research including adverse findings on risk factors, may not reach the study participants and communities. The researchers in the local context may also lose career benefits that should have accrued to them since they have been involved in the setting up of the original studies. Often, one reason for data export is lack of local capacity to analyze the samples, although African biorepositories have been attested to be able to collect, process, store, and ship biospecimens of good quality [25]. One way of addressing this issue, beyond strict enforcement of MTAs, will be to improve the capacity of researchers in Africa and provide the necessary logistics including reagents and equipment, so that most analysis of biospecimens would be done locally [28]. Other times the export is necessitated because the partner institutions also have their own larger biorepositories [29].

Biobanks and genomic data may provide public benefits as information obtained from these studies could be beneficial to pharmaceutical companies. However, there may not be direct individual benefits. Issues of individual compensation have been encountered though most individual participants advocate the public good [9].

Unfortunately, although one scoping review found as many as thirty-six guidelines or policy documents for biobanking or genomic studies within the African context, our included studies did not report participants referring to any of these guidelines [30]. Such frameworks must necessarily empower local scientists based in Africa to spearhead genomic research and biobanking in the jurisdiction. Continuous capacity building in bioethics, data analyses and bioinformatics, with these guidelines as references, would be required. National RECs must also work together in this endeavor of harmonizing ethical reviews at all levels and across institutions or organizations. This will help to harmonize existing guidelines or policy documents. In some settings such as Ghana, it is commonly known that even at national levels RECs work independently of each other, with sometimes researchers required to get approvals from multiple RECs before they carry out their work [31].

There is an urgent need to establish robust ethics frameworks and governance systems that will ensure that biobanking and genomic research in Africa is anchored on systems that allow participants derive optimum benefits from any shared data. A best practice guideline such as the H3 Africa ethical framework on biobanking and genomic research is highly recommended [25]. It draws on existing policy documents and empowers African researchers and communities, educating them on their rights and demand greater control over sample collection, storage and usage, and also deals with rules of engagement for collaborating and funding non-African institutions that they work with [32].

## Strengths and limitations

This narrative synthesis reviewed literature from a wide range of databases using a systematic approach, and we believe it provides comprehensive data from relevant publications based on our scope. We however envisage some methodological limitations with our work. First, we admit that the African region produces other language publications such as French and Portuguese, and our restriction to publications in English language may be a limitation. We however believe our search was very comprehensive with inclusion of current literature through up to 2020. Lastly due to the nature of biobanking and the evident lack of knowledge about its significance among communities, it is possible that participants in these studies may not represent the true populations, with some segments of the populations being under-reported in studies.

## Conclusion

Biobanking and genomic studies are a real need in Africa and are increasing numbers, despite the poor knowledge levels of communities on the subject. Related to this are ethical considerations related to setting up and participation in biobanks as well as data storage, export, use and sharing. Although this review shows evidence from a few African countries, it shows there is emerging or pre-existing consensus around the acceptability of broad consent as a suitable model of consent, the need for Africans to take the lead in international collaborative studies, with deliberate efforts to build capacity in local storage and analysis of samples and employ processes of sample collection and use that build trust of communities and potential study participants. Research ethics committees, researchers (including international collaborators), communities and individuals have roles to play in these Expertise of RECs in Africa especially should be built for efficient regulation and governance of biobanks and genomic research, There is also the need for more empirical studies on well-thought-out governance structures that would address the persisting ethical concerns and considerations for conducting genomics research in Africa. The careful adoption and implementation of the framework for the governance of biobanking and genomics research developed by the H3Africa, or other similar frameworks is recommended as a first step towards a more coherent and impactful governance regime for biobanking and genomic research in Africa.

### Supplementary Information

**Additional file 1.** Search String.

## Data Availability

All data sources for this review are available through the search string provided in Additional File 1. The included studies are referenced in this paper and available for readers. The information on quality assessment is duly provided in the text. Authors are available to provide any further information on assessing any of the data refereed to in the manuscript, and can be contacted through the corresponding author.

## Abbreviations

ICF: Informed consent form
MTA: Material transfer agreements
NYU: New York University
REC: Review and ethics committees
SOPs: Standard operating procedures

## References

[1] Levy SE, Myers RM (2016). Advancements in Next-Generation Sequencing. Annu Rev Genomics Hum Genet.

[2] Ahmed E, Shabani M. DNA Data Marketplace: An Analysis of the Ethical Concerns Regarding the Participation of the Individuals. Frontiers in Genetics. 2019 ;10. Available from:https://www.frontiersin.org/articles/10.3389/fgene.2019.01107. Cited 2023 Jul 12. 10.3389/fgene.2019.01107 PMC6844291 31749843

[3] Takashima K, Maru Y, Mori S, Mano H, Noda T, Muto K (2018). Ethical concerns on sharing genomic data including patients’ family members. BMC Med Ethics.

[4] Oliver JM, Slashinski MJ, Wang T, Kelly PA, Hilsenbeck SG, McGuire AL (2011). Balancing the Risks and Benefits of Genomic Data Sharing: Genome Research Participants’ Perspectives. Public Health Genomics.

[5] Tindana P, Yakubu A, Staunton C, Matimba A, Littler K, Madden E (2019). Engaging research ethics committees to develop an ethics and governance framework for best practices in genomic research and biobanking in Africa: the H3Africa model. BMC Med Ethics.

[6] Tindana P, de Vries J (2016). Broad Consent for Genomic Research and Biobanking: Perspectives from Low- and Middle-Income Countries. Annu Rev Genomics Hum Genet.

[7] Shamseer L, Moher D, Clarke M, Ghersi D, Liberati A, Petticrew M (2015). Preferred reporting items for systematic review and meta-analysis protocols (PRISMA-P) 2015: elaboration and explanation. BMJ.

[8] Akinyemi RO, Owolabi MO, Oyeniyi T, Ovbiagele B, Arnett DK, Tiwari HK (2016). Neurogenomics in Africa: Perspectives, progress, possibilities and priorities. J Neurol Sci.

[9] Abdelhafiz AS, Sultan EA, Ziady HH, Ahmed E, Khairy WA, Sayed DM (2019). What Egyptians think. Knowledge, attitude, and opinions of Egyptian patients towards biobanking issues. BMC Medical Ethics..

[10] O’Neill S, Dierickx S, Okebe J, Dabira E, Gryseels C, d’Alessandro U (2016). The Importance of Blood Is Infinite: Conceptions of Blood as Life Force, Rumours and Fear of Trial Participation in a Fulani Village in Rural Gambia. PLoS ONE.

[11] Van Schalkwyk G, de Vries J, Moodley K (2012). “It’s for a good cause, isn’t it?” - Exploring views of South African TB research participants on sample storage and re-use. BMC Med Ethics.

[12] Tindana P, Bull S, Amenga-Etego L, de Vries J, Aborigo R, Koram K (2012). Seeking consent to genetic and genomic research in a rural Ghanaian setting: A qualitative study of the MalariaGEN experience. BMC Med Ethics.

[13] Moodley K, Singh S (2016). “It’s all about trust”: reflections of researchers on the complexity and controversy surrounding biobanking in South Africa. BMC Med Ethics.

[14] Tindana P, Molyneux CS, Bull S, Parker M (2014). Ethical issues in the export, storage and reuse of human biological samples in biomedical research: perspectives of key stakeholders in Ghana and Kenya. BMC Med Ethics.

[15] Igbe MA, Adebamowo CA (2012). Qualitative study of knowledge and attitudes to biobanking among lay persons in Nigeria. BMC Med Ethics.

[16] Mwaka E, Horn L (2019). Researchers’ Perspectives on Informed Consent and Ethical Review of Biobank Research in South Africa: A Cross-Sectional Study. J Empir Res Hum Res Ethics.

[17] Wendler D, Pace C, Talisuna AO, Maiso F, Grady C, Emanuel E (2005). Research on stored biological samples: the views of Ugandans. IRB.

[18] Barchi F, Matlhagela K, Jones N, Kebaabetswe PM, Merz JF (2015). “The keeping is the problem”: A qualitative study of IRB-member perspectives in Botswana on the collection, use, and storage of human biological samples for research. BMC Med Ethics.

[19] Moodley K, Sibanda N, February K, Rossouw T (2014). “It’s my blood”: ethical complexities in the use, storage and export of biological samples: perspectives from South African research participants. BMC Med Ethics.

[20] World Medical Association (2013). World Medical Association Declaration of Helsinki: Ethical Principles for Medical Research Involving Human Subjects. JAMA.

[21] Husedzinovic A, Ose D, Schickhardt C, Fröhling S, Winkler EC (2015). Stakeholders’ perspectives on biobank-based genomic research: systematic review of the literature. Eur J Hum Genet.

[22] Juengst ET, Meslin EM (2019). Sharing with Strangers: Governance Models for Borderless Genomic Research in a Territorial World. Kennedy Inst Ethics J.

[23] Staunton C, Tindana P, Hendricks M, Moodley K (2018). Rules of engagement: perspectives on stakeholder engagement for genomic biobanking research in South Africa. BMC Med Ethics.

[24] Bukini D, deVries J, Treadwell M, Anie K, Dennis-Antwi J, Kamga KK (2019). Exploring the Role of Shared Decision Making in the Consent Process for Pediatric Genomics Research in Cameroon, Tanzania, and Ghana. AJOB Empirical Bioethics.

[25] Yakubu A, Munung NS, Vries JD (2020). How Should Biobanking Be Governed in Low-Resource Settings?. AMA J Ethics.

[26] Wonkam A, de Vries J (2020). Returning incidental findings in African genomics research. Nat Genet.

[27] Lewis ACF, Knoppers BM, Green RC (2021). An international policy on returning genomic research results. Genome Med.

[28] Owusu SA, Addison G, Redman B, Kearns L, Amuna P, Laar A (2022). Assessment of the Operational Characteristics of Research Ethics Committees in Ghana. J Empir Res Hum Res Ethics.

[29] Croxton T, Swanepoel C, Musinguzi H, Kader M, Ozumba P, Pillay AD (2017). Lessons Learned from Biospecimen Shipping Among the Human Heredity and Health in Africa Biorepositories. Biopreserv Biobank.

[30] Ali J, Cohn B, Mwaka E, Bollinger JM, Kwagala B, Barugahare J (2021). A scoping review of genetics and genomics research ethics policies and guidelines for Africa. BMC Med Ethics.

[31] Owusu SA. Ethical implications of the widespread use of informal mHealth methods in Ghana. J Med Ethics. 2022 Apr 8:medethics-2021–107920. doi: 10.1136/medethics-2021-107920 Epub ahead of print. PMID: 35396337; PMCID: PMC9547032. 10.1136/medethics-2021-107920 PMC9547032 35396337

[32] A welcome framework for research in Africa. Nature. 2018;556(7701):274–274. 10.1038/d41586-018-04589-0 29670269

