# Supplementary material for: Ethical considerations for biobanking and use of genomics data in Africa: a narrative review
Source: BMC Med Ethics. 2023 Dec 5;24:108. doi: 10.1186/s12910-023-00985-y (PMC10699036; doi:10.1186/s12910-023-00985-y)
Supplement: Supplementary file 1 — Additional file 1. Search String. [file 12910_2023_985_MOESM1_ESM.pdf]

Additional File 1: Search String

Cochrane

((biobank or biobanking or biological specimen banks or biomedical research or specimen handling or genomics) and (research ethics or ethics or ethical or ethics research or research ethics committee) and (Africa or African or Algeria or Angola or Benin or Botswana or Burkina Faso or Burundi or Cabo Verde or Cameroon or Cameroun or Cape Verde or Central African Republic or Chad or Comoros or Congo or Cote d'Ivoire or Ivory Coast Democratic Republic of Congo or Djibouti or Egypt or Eritrea or eSwatini or Ethiopia or Gabon or Gambia or Ghana or Guinea or Guinea- Bissau or Jamahiriya or Kenya or Lesotho or Liberia or Libya or Madagascar or Malawi or Mali or Mauritania or Mauritius or Mayotte or Morocco or Mozambique or Namibia or Niger or Nigeria or Principe or Reunion or Rwanda or Saint Helena or Sao Tome or Senegal or Seychelles or Sierra Leone or Somalia or Sudan or Swaziland or Tanzania or Togo or Tunisia or Uganda or Zaire or Zambia or Zimbabwe or Sub-Saharan Africa or Central Africa or Eastern Africa or Southern Africa or Western Africa or Northern Africa)).mp. [mp=ti, ab, tx, kw, ct, ot, sh, hw] (45)

PubMed

(("biobank"[All Fields] OR "biobanked"[All Fields] OR "biobankers"[All Fields] OR "biobanking"[All Fields] OR "biobanks"[All Fields] OR ("biobank"[All Fields] OR "biobanked"[All Fields] OR "biobankers"[All Fields] OR "biobanking"[All Fields] OR "biobanks"[All Fields]) OR ("biological specimen banks"[MeSH Terms] OR ("biological"[All Fields] AND "specimen"[All Fields] AND "banks"[All Fields]) OR "biological specimen banks"[All Fields]) OR ("biomedical research"[MeSH Terms] OR ("biomedical"[All Fields] AND "research"[All Fields]) OR "biomedical research"[All Fields]) OR ("specimen handling"[MeSH Terms] OR ("specimen"[All Fields] AND "handling"[All Fields]) OR "specimen handling"[All Fields]) OR ("genome"[MeSH Terms] OR "genome"[All Fields] OR "genomes"[All Fields] OR "genome s"[All Fields] OR "genomically"[All Fields] OR "genomics"[MeSH Terms] OR "genomics"[All Fields] OR "genomic"[All Fields])) AND ("ethics, research"[MeSH Terms] OR ("ethics"[All Fields] AND "research"[All Fields]) OR "research ethics"[All Fields] OR ("research"[All Fields] AND "ethics"[All Fields]) OR ("ethic s"[All Fields] OR "ethicality"[All Fields] OR "ethically"[All Fields] OR "ethics"[MeSH Terms] OR "ethics"[All Fields] OR "ethic"[All Fields] OR "ethics"[MeSH Subheading] OR "morals"[MeSH Terms] OR "morals"[All Fields] OR "ethical"[All Fields]) OR ("ethic s"[All Fields] OR "ethicality"[All Fields] OR "ethically"[All Fields] OR "ethics"[MeSH Terms] OR "ethics"[All Fields] OR "ethic"[All Fields] OR "ethics"[MeSH Subheading] OR "morals"[MeSH Terms] OR "morals"[All Fields] OR "ethical"[All Fields]) OR ("ethics, research"[MeSH Terms] OR ("ethics"[All Fields] AND "research"[All Fields]) OR "research ethics"[All Fields] OR ("ethics"[All Fields] AND "research"[All Fields]) OR "ethics research"[All Fields]) OR ("ethics committees, research"[MeSH Terms] OR ("ethics"[All Fields] AND "committees"[All Fields] AND "research"[All Fields]) OR "research ethics committees"[All Fields] OR ("research"[All Fields] AND "ethics"[All Fields] AND "committee"[All Fields]) OR "research ethics committee"[All Fields])) AND ("africa"[MeSH Terms] OR "africa"[All Fields] OR "africa s"[All Fields] OR "africas"[All Fields] OR ("african continental ancestry group"[MeSH Terms] OR ("african"[All Fields] AND "continental"[All Fields] AND "ancestry"[All Fields] AND "group"[All Fields]) OR "african continental ancestry group"[All Fields] OR "african"[All Fields] OR "africans"[All Fields]) OR ("algeria"[MeSH Terms] OR "algeria"[All Fields]) OR ("angola"[MeSH Terms] OR "angola"[All Fields] OR "angola s"[All Fields]) OR ("benin"[MeSH Terms] OR "benin"[All Fields] OR "benin s"[All Fields])

OR ("botswana"[MeSH Terms] OR "botswana"[All Fields] OR "botswana s"[All Fields]) OR ("burkina faso"[MeSH Terms] OR ("burkina"[All Fields] AND "faso"[All Fields]) OR "burkina faso"[All Fields]) OR ("burundi"[MeSH Terms] OR "burundi"[All Fields]) OR ("cabo verde"[MeSH Terms] OR ("cabo"[All Fields] AND "verde"[All Fields]) OR "cabo verde"[All Fields]) OR ("cameroon"[MeSH Terms] OR "cameroon"[All Fields] OR "cameroons"[All Fields] OR "cameroon s"[All Fields]) OR "Cameroun"[All Fields] OR ("cabo verde"[MeSH Terms] OR ("cabo"[All Fields] AND "verde"[All Fields]) OR "cabo verde"[All Fields] OR ("cape"[All Fields] AND "verde"[All Fields]) OR "cape verde"[All Fields]) OR ("central african republic"[MeSH Terms] OR ("central"[All Fields] AND "african"[All Fields] AND "republic"[All Fields]) OR "central african republic"[All Fields]) OR ("chad"[MeSH Terms] OR "chad"[All Fields]) OR ("comoros"[MeSH Terms] OR "comoros"[All Fields] OR "comoro"[All Fields]) OR ("congo"[MeSH Terms] OR "congo"[All Fields]) OR ("cote d ivoire"[MeSH Terms] OR ("cote"[All Fields] AND "d ivoire"[All Fields]) OR "cote d ivoire"[All Fields]) OR (("cote d ivoire"[MeSH Terms] OR ("cote"[All Fields] AND "d ivoire"[All Fields]) OR "cote d ivoire"[All Fields] OR ("ivory"[All Fields] AND "coast"[All Fields]) OR "ivory coast"[All Fields]) AND ("democrat"[All Fields] OR "democratic"[All Fields] OR "democratically"[All Fields] OR "democratization"[All Fields] OR "democratize"[All Fields] OR "democratized"[All Fields] OR "democratizing"[All Fields] OR "democrats"[All Fields]) AND ("republic"[All Fields] OR "republic s"[All Fields] OR "republics"[All Fields]) AND ("congo"[MeSH Terms] OR "congo"[All Fields])) OR ("djibouti"[MeSH Terms] OR "djibouti"[All Fields]) OR ("egypt"[MeSH Terms] OR "egypt"[All Fields] OR "egypt s"[All Fields]) OR ("eritrea"[MeSH Terms] OR "eritrea"[All Fields]) OR ("eswatini"[MeSH Terms] OR "eswatini"[All Fields]) OR ("ethiopia"[MeSH Terms] OR "ethiopia"[All Fields] OR "ethiopia s"[All Fields]) OR ("gabon"[MeSH Terms] OR "gabon"[All Fields]) OR ("gambia"[MeSH Terms] OR "gambia"[All Fields] OR "gambia s"[All Fields]) OR ("ghana"[MeSH Terms] OR "ghana"[All Fields] OR "ghana s"[All Fields]) OR ("guinea"[MeSH Terms] OR "guinea"[All Fields] OR "guinea s"[All Fields] OR "guineas"[All Fields]) OR ("guinea bissau"[MeSH Terms] OR "guinea bissau"[All Fields] OR ("guinea"[All Fields] AND "bissau"[All Fields]) OR "guinea bissau"[All Fields]) OR "Jamahiriya"[All Fields] OR ("kenya"[MeSH Terms] OR "kenya"[All Fields] OR "kenya s"[All Fields]) OR ("lesotho"[MeSH Terms] OR "lesotho"[All Fields]) OR ("liberia"[MeSH Terms] OR "liberia"[All Fields] OR "liberia s"[All Fields]) OR ("libya"[MeSH Terms] OR "libya"[All Fields]) OR ("madagascar"[MeSH Terms] OR "madagascar"[All Fields] OR "madagascar s"[All Fields]) OR ("malawi"[MeSH Terms] OR "malawi"[All Fields] OR "malawi s"[All Fields]) OR ("mali"[MeSH Terms] OR "mali"[All Fields]) OR ("mauritania"[MeSH Terms] OR "mauritania"[All Fields]) OR ("mauritius"[MeSH Terms] OR "mauritius"[All Fields]) OR ("comoros"[MeSH Terms] OR "comoros"[All Fields] OR "mayotte"[All Fields]) OR ("morocco"[MeSH Terms] OR "morocco"[All Fields]) OR ("mozambique"[MeSH Terms] OR "mozambique"[All Fields] OR "mozambique s"[All Fields]) OR ("namibia"[MeSH Terms] OR "namibia"[All Fields]) OR ("niger"[MeSH Terms] OR "niger"[All Fields]) OR ("nigeria"[MeSH Terms] OR "nigeria"[All Fields] OR "nigeria s"[All Fields]) OR ("principe"[All Fields] OR "principes"[All Fields]) OR ("reunion"[MeSH Terms] OR "reunion"[All Fields] OR "reunions"[All Fields]) OR ("rwanda"[MeSH Terms] OR "rwanda"[All Fields] OR "rwanda s"[All Fields]) OR ("atlantic islands"[MeSH Terms] OR ("atlantic"[All Fields] AND "islands"[All Fields]) OR "atlantic islands"[All Fields] OR ("saint"[All Fields] AND "helena"[All Fields]) OR "saint helena"[All Fields]) OR ("Sao"[All Fields] AND "Tome"[All Fields]) OR ("senegal"[MeSH Terms] OR "senegal"[All Fields] OR "senegal s"[All Fields]) OR ("seychelles"[MeSH Terms] OR "seychelles"[All Fields]) OR ("sierra leone"[MeSH Terms] OR ("sierra"[All Fields] AND "leone"[All Fields]) OR "sierra leone"[All Fields]) OR

("somalia"[MeSH Terms] OR "somalia"[All Fields]) OR ("sudan"[MeSH Terms] OR "sudan"[All Fields] OR "sudans"[All Fields] OR "sudan s"[All Fields]) OR ("eswatini"[MeSH Terms] OR "eswatini"[All Fields] OR "swaziland"[All Fields]) OR ("tanzania"[MeSH Terms] OR "tanzania"[All Fields] OR "tanzania s"[All Fields]) OR ("togo"[MeSH Terms] OR "togo"[All Fields]) OR ("tunisia"[MeSH Terms] OR "tunisia"[All Fields]) OR ("uganda"[MeSH Terms] OR "uganda"[All Fields] OR "uganda s"[All Fields]) OR ("democratic republic of the congo"[MeSH Terms] OR ("democratic"[All Fields] AND "republic"[All Fields] AND "congo"[All Fields]) OR "democratic republic of the congo"[All Fields] OR "zaire"[All Fields]) OR ("zambia"[MeSH Terms] OR "zambia"[All Fields] OR "zambia s"[All Fields]) OR ("zimbabwe"[MeSH Terms] OR "zimbabwe"[All Fields] OR "zimbabwe s"[All Fields]) OR ("africa south of the sahara"[MeSH Terms] OR ("africa"[All Fields] AND "south"[All Fields] AND "sahara"[All Fields]) OR "africa south of the sahara"[All Fields] OR ("sub"[All Fields] AND "saharan"[All Fields] AND "africa"[All Fields]) OR "sub saharan africa"[All Fields]) OR ("africa, central"[MeSH Terms] OR ("africa"[All Fields] AND "central"[All Fields]) OR "central africa"[All Fields] OR ("central"[All Fields] AND "africa"[All Fields])) OR ("africa, eastern"[MeSH Terms] OR ("africa"[All Fields] AND "eastern"[All Fields]) OR "eastern africa"[All Fields] OR ("eastern"[All Fields] AND "africa"[All Fields])) OR ("africa, southern"[MeSH Terms] OR ("africa"[All Fields] AND "southern"[All Fields]) OR "southern africa"[All Fields] OR ("southern"[All Fields] AND "africa"[All Fields])) OR ("africa, western"[MeSH Terms] OR ("africa"[All Fields] AND "western"[All Fields]) OR "western africa"[All Fields] OR ("western"[All Fields] AND "africa"[All Fields])) OR ("africa, northern"[MeSH Terms] OR ("africa"[All Fields] AND "northern"[All Fields]) OR "northern africa"[All Fields] OR ("northern"[All Fields] AND "africa"[All Fields]))) AND "humans"[MeSH Terms]) NOT ("African Americans"[MeSH Terms] OR "African Americans"[All Fields])

Embase

('prevalence'/exp OR prevalence OR 'diabetes prevalence') AND ('diabetes mellitus'/exp OR 'diabetes mellitus' OR 'diabetes'/exp OR 'diabetes' OR 'type 2 diabetes mellitus'/exp OR 'type 2 diabetes mellitus') AND ('risk factor'/exp OR 'risk factor' OR 'risk factors'/exp OR 'risk factors' OR determinants) AND ('ghana'/exp OR ghana OR 'ghanaian'/exp OR ghanaian) AND [humans]/lim ((biobank or biobanking or biological specimen banks or biomedical research or specimen handling or genomics) and (research ethics or ethics or ethical or ethics research or research ethics committee) and (Africa or African or Algeria or Angola or Benin or Botswana or Burkina Faso or Burundi or Cabo Verde or Cameroon or Cameroun or Cape Verde or Central African Republic or Chad or Comoros or Congo or Cote d'Ivoire or Ivory Coast Democratic Republic of Congo or Djibouti or Egypt or Eritrea or eSwatini or Ethiopia or Gabon or Gambia or Ghana or Guinea or Guinea- Bissau or Jamahiriya or Kenya or Lesotho or Liberia or Libya or Madagascar or Malawi or Mali or Mauritania or Mauritius or Mayotte or Morocco or Mozambique or Namibia or Niger or Nigeria or Principe or Reunion or Rwanda or Saint Helena or Sao Tome or Senegal or Seychelles or Sierra Leone or Somalia or Sudan or Swaziland or Tanzania or Togo or Tunisia or Uganda or Zaire or Zambia or Zimbabwe or Sub-Saharan Africa or Central Africa or Eastern Africa or Southern Africa or Western Africa or Northern Africa)).mp. [mp=title, abstract, heading word, table of contents, key concepts, original title, tests & measures, mesh] (118)

Cinahl

(biobank OR biobanking OR biological specimen banks OR biomedical research OR specimen handling OR genomics) AND (research ethics OR ethics OR ethical OR ethics research OR research ethics committee) AND (Africa OR African OR Algeria OR Angola OR Benin OR Botswana OR Burkina Faso OR Burundi OR Cabo Verde OR Cameroon OR

Cameroun OR Cape Verde OR Central African Republic OR Chad OR Comoros OR Congo OR Cote d'Ivoire OR Ivory Coast Democratic Republic of Congo OR Djibouti OR Egypt OR Eritrea OR eSwatini OR Ethiopia OR Gabon OR Gambia OR Ghana OR Guinea OR Guinea-Bissau OR Jamahiriya OR Kenya OR Lesotho OR Liberia OR Libya OR Madagascar OR Malawi OR Mali OR Mauritania OR Mauritius OR Mayotte OR Morocco OR Mozambique OR Namibia OR Niger OR Nigeria OR Principe OR Reunion OR Rwanda OR Saint Helena OR Sao Tome OR Senegal OR Seychelles OR Sierra Leone OR Somalia OR Sudan OR Swaziland OR Tanzania OR Togo OR Tunisia OR Uganda OR Zaire OR Zambia OR Zimbabwe OR Sub-Saharan Africa OR Central Africa OR Eastern Africa OR Southern Africa OR Western Africa OR Northern Africa)

Web of Science OR SCIELO OR BCI

You searched for: TOPIC:((biobank OR biobanking OR biological specimen banks OR biomedical research OR specimen handling OR genomics) AND (research ethics OR ethics OR ethical OR ethics research OR research ethics committee) AND (Africa OR African OR Algeria OR Angola OR Benin OR Botswana OR Burkina Faso OR Burundi OR Cabo Verde OR Cameroon OR Cameroun OR Cape Verde OR Central African Republic OR Chad OR Comoros OR Congo OR Cote d'Ivoire OR Ivory Coast Democratic Republic of Congo OR Djibouti OR Egypt OR Eritrea OR eSwatini OR Ethiopia OR Gabon OR Gambia OR Ghana OR Guinea OR Guinea-Bissau OR Jamahiriya OR Kenya OR Lesotho OR Liberia OR Libya OR Madagascar OR Malawi OR Mali OR Mauritania OR Mauritius OR Mayotte OR Morocco OR Mozambique OR Namibia OR Niger OR Nigeria OR Principe OR Reunion OR Rwanda OR Saint Helena OR Sao Tome OR Senegal OR Seychelles OR Sierra Leone OR Somalia OR Sudan OR Swaziland OR Tanzania OR Togo OR Tunisia OR Uganda OR Zaire OR Zambia OR Zimbabwe OR Sub-Saharan Africa OR Central Africa OR Eastern Africa OR Southern Africa OR Western Africa OR Northern Africa))
